# HandyCNV: Standardized Summary, Annotation, Comparison, and Visualization of Copy Number Variant, Copy Number Variation Region, and Runs of Homozygosity

**DOI:** 10.3389/fgene.2021.731355

**Published:** 2021-09-17

**Authors:** Jinghang Zhou, Liyuan Liu, Thomas J. Lopdell, Dorian J. Garrick, Yuangang Shi

**Affiliations:** ^1^School of Agriculture, Ningxia University, Yinchuan, China; ^2^AL Rae Centre for Genetics and Breeding, Massey University, Hamilton, New Zealand; ^3^Research and Development, Livestock Improvement Corporation, Hamilton, New Zealand

**Keywords:** copy number variant, run of homozygosity, haplotype, SNP, CNVR

## Abstract

Detection of CNVs (copy number variants) and ROH (runs of homozygosity) from SNP (single nucleotide polymorphism) genotyping data is often required in genomic studies. The post-analysis of CNV and ROH generally involves many steps, potentially across multiple computing platforms, which requires the researchers to be familiar with many different tools. In order to get around this problem and improve research efficiency, we present an R package that integrates the summarization, annotation, map conversion, comparison and visualization functions involved in studies of CNV and ROH. This one-stop post-analysis system is standardized, comprehensive, reproducible, timesaving, and user-friendly for researchers in humans and most diploid livestock species.

## Introduction

Genome-wide data have been accumulated for large numbers of individuals of various species as the cost of single nucleotide polymorphism (SNP) genotyping continues to decrease. In addition to using these data for GWAS (genome wide association study) or GS (genomic selection), interesting genomic information about copy number variant (CNV) and runs of homozygosity (ROH) can be inferred from these genotypes, and a range of software products [such as PennCNV (Wang et al., 2007), CNVPartition (Illumina, 2021), SNP and Variation Suite (Bozeman and Golden Helix, 2020)] have been developed to detect CNV and ROH for SNP data. However, few tools can integrate the summary data with annotations, comparisons, and visualizations of these results. As a result, extracting useful information from CNV and ROH data sets is time consuming, especially when it requires processing multiple results from different models and software. In order to get more comprehensive results, researchers often implement their own pipelines to switch back and forth between different tools, an approach that is prone to introducing bugs and thereby producing spurious results.

There are several common “pitfalls” we have observed when conducting CNV analyses using SNP genotyping data. The most frequent is to annotate the candidate genes in a CNVR (copy number variation region) without considering the frequency of the CNVs: this can result in undue weight being given to rare CNVs that affect only one or two samples. A second issue is comparing CNVs between different studies, and making comparisons only at the population level, and not at the individual sample level. Comparison at the population level could reflect the ubiquitous nature of CNVs, but at the individual level it also provides information about the robustness of CNV detection algorithms. A third issue arises when comparing CNVRs that have been detected using different reference genomes, which requires converting the coordinates of the regions between the two genomes. Making these conversions requires careful consideration, as the order of SNPs on chromosomes might differ between two different reference assemblies, such that the lengths or even chromosomal orders of CNVs can change, which might lead to meaningless comparisons between CNVRs. A fourth common problem is get the incorrect number of overlapping CNVRs when presenting comparison results via Venn diagram. Since the number of overlapping regions is relative to the results, and a single long interval generated using one approach might overlap multiple shorter intervals detected using another approach, in which case representing the results via Venn diagram requires special annotation.

There are also some steps that may be easily forgotten performing ROH analysis on SNP genotyping data. For example, the SNP density distributions may not have been carefully examined prior to inference of ROH. The density of SNPs may differ across the chromosome on different SNP chips, but ROH detection methods are highly affected by characteristics such as SNP density, window size, tolerance of occasional heterozygosity in the run, and the presence of missing values in the detection window. Knowing SNP density can therefore help us to select better parameters when performing ROH detection. Moreover, while reporting the candidate genes by functional annotation of genes that located in ROH regions, we may not examine the frequencies of haplotypes within these interesting genes, but this step could provide valuable information about the high frequency genotypes of these genes, which is useful on designing the further validation experiments and can provide the valuable reference to others when they comparing the genes using the same SNP chips on different populations.

There are several common requirements in studying CNV and ROH patterns in a new species or population. These include: the need for preparing summary tables, making summary figures, generating CNVRs and plotting CNVR distribution maps with gene annotations, comparing CNVs and CNVRs between studies, converting genome coordinates and map files from one reference to another, finding high frequency abnormal genomic regions, creating consensus gene lists, producing custom visualization of results, and identifying haplotypes in regions of interest. Therefore, we built this open-source tool to provide a standardized, reproducible, time-saving and widely available one-stop post-analysis system to make research more simple, practical and efficient while avoiding common “pitfalls” that can affect the accuracy and interpretability of these studies.

## Method

### Brief Introduction of Main Functions

**FIGURE 1:**
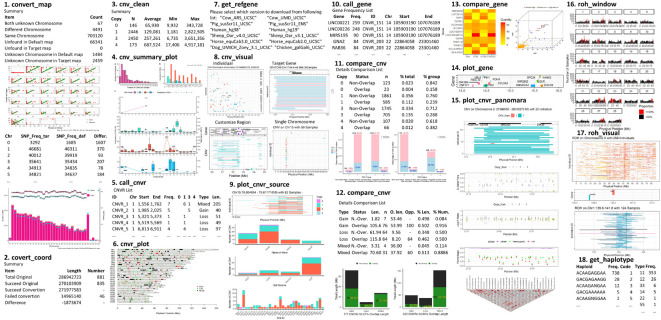
Example plots illustrating the main functions and output from the HandyCNV package.

The functions provided by this package can be categorized into five sections: Conversion; Summary; Annotation; Comparison; and Visualization. The most useful features provided are: integrating summarized results, generating lists of CNVRs, annotating the results with known gene positions, plotting CNVR distribution maps, and producing customized visualizations of CNVs and ROHs with gene and other related information on one plot (Figure 1). This package supports a range of customizations, including the color, size of high-resolution figures, and choice of output folder to avoid conflict between the results of different runs. Where applicable, output files are compatible with other software such as PennCNV (Wang et al., 2007), Plink (Chang et al., 2015), or DAVID annotation tools (Jiao et al., 2012).

The conversion section handles the conversions of genomic positions between two reference genomes, and provides two functions. *convert_map* is designed to compare SNP map files for two different reference genomes, matching by SNP name, and produce SNP maps in a format suitable for use by *convert_coord*. The function also reports the density of SNPs by chromosome. *convert_coord* is designed to convert the physical positions of genomic intervals based on a given SNP map file. Currently, the function is limited to inputs generated by *convert_map*, and can only convert the coordinates for intervals on the same type of SNP chip. Converting coordinates may change the total length of the intervals, as the positions and orders of the SNPs on the chromosome will potentially differ between various reference genomes; therefore, the function produces a table that summarizes how many intervals were converted successfully, and reports on the differences in length between the converted and original intervals.

The summary section contains a group of functions to summarize CNV results, generate CNVRs, and make CNVR distribution maps from CNV results. There is also a collection of functions to summarize ROH results, report frequencies of ROH regions, inbreeding coefficient by different length groups and to generate haplotypes on interesting ROH regions.

The functions used for reporting CNV results include *clean_cnv*, *summary_cnv_plot*, and *call_cnvr*. *clean_cnv* takes a CNV list from PennCNV and CNVPartition and reformats it into a standard format for use in the functions listed below. *cnv_summary_plot* generates a range of summary plots, aggregating CNV results by length group, CNV type, chromosome, and individual. *call_cnvr* generates CNV regions as the union of sets of CNVs that overlap by at least one base pair (Redon et al., 2006). This function will output three tables: (a) the list of CNVRs, containing the number of CNVs and number of samples in each CNVR that can reflect the frequency of CNVRs; (b) a brief summary table showing numbers of CNVRs by length and type (Deletion, Duplication, and Mixed, where Mixed indicates that both duplications and deletions are found within the CNVR); and (c) the total length and number of CNVRs on each chromosome.

*roh_window* will report: a table of high frequency ROH regions on the autosomes that passed the common frequency threshold, a table containing inbreeding coefficients by different length groups of each individual, a brief summary of the total numbers and lengths of ROHs in length groups, and a plot of high frequency ROH regions by chromosome. The inbreeding coefficients are calculated as *F*_*roh*_ = (∑*L*_*roh*_)/(∑*L*_*auto*_) (McQuillan et al., 2008), where ∑*L*_*roh*_ is the total length of ROH, and ∑*L*_*auto*_ is the total length of autosomes. Other functions in this group include *prep_phased*, *closer_snp*, and *get_haplotype*; see the package vignette for more information (Jinghang et al., 2021).

The annotation section facilitates downloading and formatting reference gene lists, and annotating genes on genomic intervals. *get_refgene* will automatically download a reference gene list and invoke *clean_ucsc* and *clean_ensgene* from UCSC (Navarro Gonzalez et al., 2021) websites for human, cow, sheep, pig, horse, chicken or dog species, then remove the duplicated genes and report the standard format as output. *call_gene* is used to report how many genes are located in the given genomic intervals. The frequency of genes is calculated from the number of samples that has the same gene annotated in its CNVs.

The comparison section consists of functions for comparing sets of CNVs (*compare_cnv*), CNVRs (*compare_cnvr*), gene frequency lists (*compare_gene*), and other intervals (*compare_interval*). These functions were implemented using the *foverlaps* function in the *data.table* R package (Dowle et al., 2019). *compare_gene* can produce consensus gene lists, given lists of genes present in CNVRs in multiple studies. The remaining functions report numbers, lengths, and proportions of overlapping intervals (CNVs, CNVRs, etc.) on a population and individual basis.

Finally, twelve functions in HandyCNV are included in the visualization section; of these, five produce plots as a subset of their output, and have been mentioned previously: *cnv_summary_plot*, *roh_window*, *compare_cnv*, *compare_cnvr*, and *convert_map*. The remaining visualization functions mainly focus on customizing and integrating the plotting of all information related to CNV, ROH, and high frequency CNVR: these are *cnvr_plot*, *plot_gene*, *cnv_visual*, *roh_visual*, *plot_cnvr_panorama*, *plot_snp_density*, and *plot_cnvr_source*. These functions are described in the package vignette (Jinghang et al., 2021).

### Pipelines for the Post Analysis of CNVs and ROHs

#### Post-analysis of CNVs and CNVRs

**FIGURE 2:**
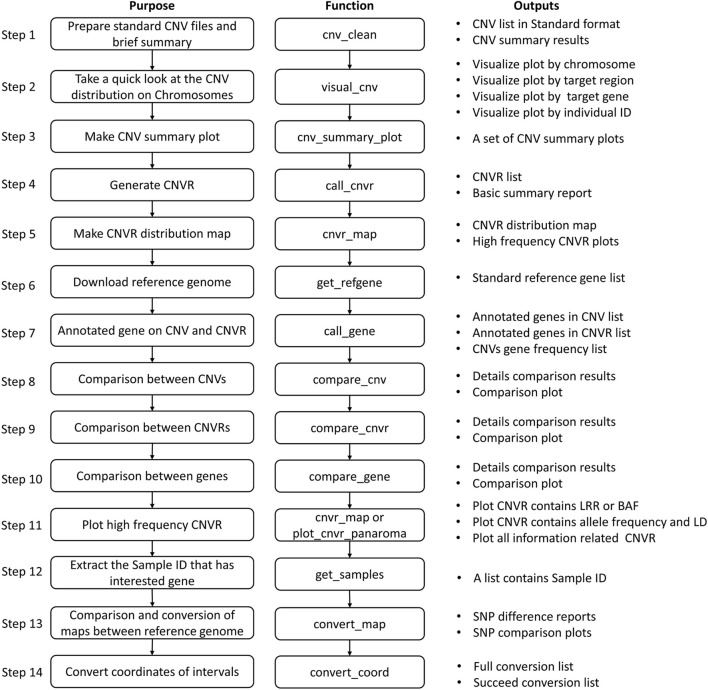
Pipeline of post analysis of CNV results using HandyCNV.

The recommended pipeline contains 14 basic steps depending on the study purposes (Figure 2), although usage is not limited to these basic steps, and users are free to explore their data by customizing the functions. By running through this pipeline, users can produce a wide range of results, such as summary tables and plots of CNV results, the CNVR list and its brief summary information and CNVR distribution plot, the frequency of CNVs and CNVRs within annotated genes, and comparison results between CNVs, CNVR, and annotated genes.

#### Post-analysis of ROHs

**FIGURE 3:**
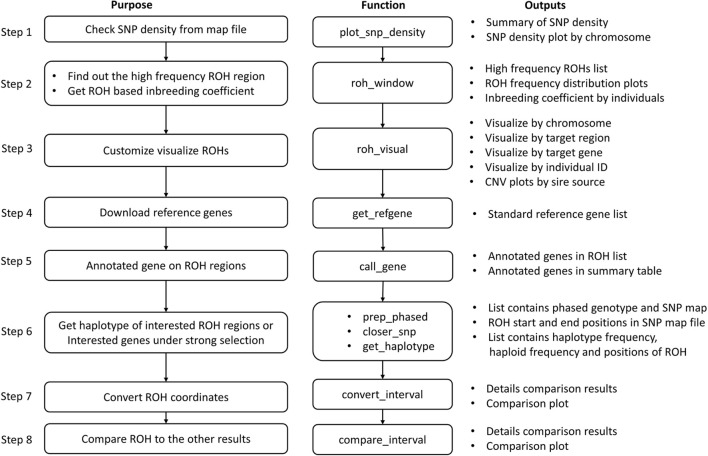
Pipeline of post-analysis of ROH in HandyCNV.

The pipeline for the post analysis of ROHs contains eight basic steps (Figure 3). The main results produced by running through this pipeline are the high frequency ROH regions list, ROH-based inbreeding coefficients, a list of genes that are located in the ROH regions, and the frequency of haplotypes within genes or regions of interest.

## Application Examples of CNV and ROH

We now provide two example runs of the pipeline, using two previously published data sets: the first is a CNV list produced for a human population in Brazil (de Godoy et al., 2020), and the second is genotype data for an inbred breed of horses (Velie et al., 2016). The purpose of these examples is to introduce how to use the functions in this package; therefore, further interpretation of the results is not included.

### Example 1. the Post-analysis of CNVs in a Human Dataset

**FIGURE 4:**
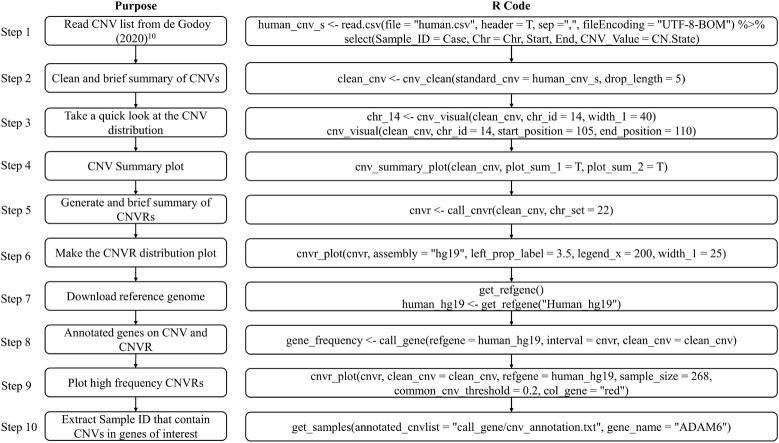
Analytical steps of example 1.

The CNV result in this example was cited from a study published in 2020 which comprised 268 microarrays samples in a human population in Brazil (de Godoy et al., 2020). In this example, we will introduce how to prepare the standard CNV list, then produce brief summary, generate CNVRs, annotate genes and visualize CNVs. Figure 4 presents the code used in example 1, the R script can be found in Supplementary File 1.

**FIGURE 5:**
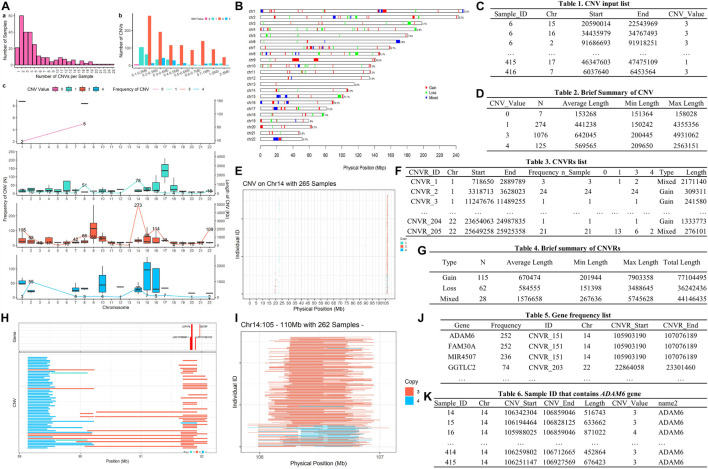
The main outputs of example 1. Panel **(A)** is CNV summary plot; panel **(B)** is CNVR distribution map; panel **(C)** is CNV input list; panel **(D)** is the brief summary table of CNV; panel **(E)** is a plot of CNVs on Chromosome 14; panel **(F)** is CNVR list; panel **(G)** is the brief summary table of CNVRs; panel **(H)** is an example plot of the high frequency CNVR; panel **(I)** is a plot of CNVs on Chr14:105-110 Mb; panel **(J)** is the gene frequency list; and panel **(K)** is the sample list that contain CNVs in the LINC00221 gene.

To replicate this example, we first need to download the dataset “Table S1 – Detailed information about all CNVs analyzed in our sample” (de Godoy et al., 2020) and save the sheet “All array platforms’ CNVs” as.*csv* format file. Then use *read.csv* to load the CNV list and select the columns required by *cnv_clean* (see Figure 5C).

A formatted clean CNV list will return as an object named “clean_cnv” in working environment, and a brief summary table of CNV (see Figure 5D) will be written out after executing *cnv_clean*.

We then take a quick look at the CNV distribution by reading the “clean_cnv” list as input and customizing parameters in *cnv_visual*. In example, we first set “chr_id = 14” to visualize CNVs distribution on chromosome 14 (see Figure 5E), then zoom into the region with higher frequency CNVs (see Figure 5I) by setting “start_position = 105” and “end_position = 110.” Visualizing other chromosomes or regions and changing the colors of copy numbers can easily be done by adjusting the relevant arguments.

The CNV summary plot (see Figure 5A) can be plotted via *cnv_summary_plot* by taking “clean_cnv” as input. The CNVR list (see Figure 5F) is generated using call_cnvr by taking the “clean_cnv” file as input, producing a brief summary table of CNVR (see Figure 5G) that will be saved in the working directory in the meantime. The CNVR distribution map (see Figure 5B) is generated via *cnvr_plot* by loading the CNVR list.

For gene annotation steps, the reference gene list can be downloaded and formatted by assigning the genome version argument in *get_refgene*. Then the genes annotation list of CNV or CNVR are generated by running *call_gene*. Three input files need be assigned in the function: the clean CNV file (“clean_cnv”), the CNVR list (“cnvr”), and the reference gene list (“human_hg19”); the gene frequency list (see Figure 5J) will be returned as an object in the R environment. We can plot all the high frequency CNVRs with gene annotation results (see one example plot in Figure 5H) at the same time through cnvr_plot by reading “cnvr,” “clean_cnv” and reference gene list (“human_hg19”) and setting the “sample_size” and “common_cnv_threshold” arguments.

Finally, we can extract Sample IDs of CNVs that contain genes of interest (see Figure 5K) using *get_samples*, by loading the CNV annotation list generated by *call_gene* and assigning the gene name to the “gene_name” argument.

Since this example only contains one CNV result in one reference genome, the functions in the comparison and conversion sections are not applicable in this example. Users of these functions can browse the vignette of this package from the Github repository (Jinghang et al., 2021).

### Example 2. the Post-analysis of ROH Using Horse Genotype Samples

**FIGURE 6:**
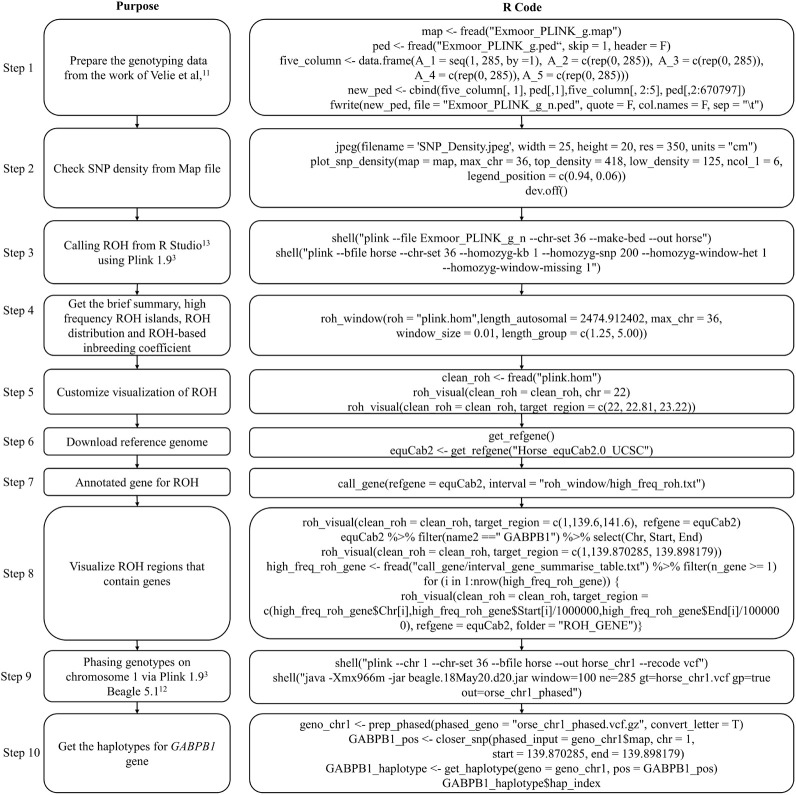
Analytical steps of example 2.

The genotype data used to detect ROH in this example is from the work of Velie et al. (2016) and contains 285 horse samples. This example aims to present how to use the functions in HandyCNV to analyze ROHs. This example includes ROH detection by Plink 1.9 (Chang et al., 2015) and genotype phasing by Beagle 5.1 (Browning et al., 2018). Figure 6 presents the code used in example 2; the R script can be found in Supplementary File 2.

**FIGURE 7:**
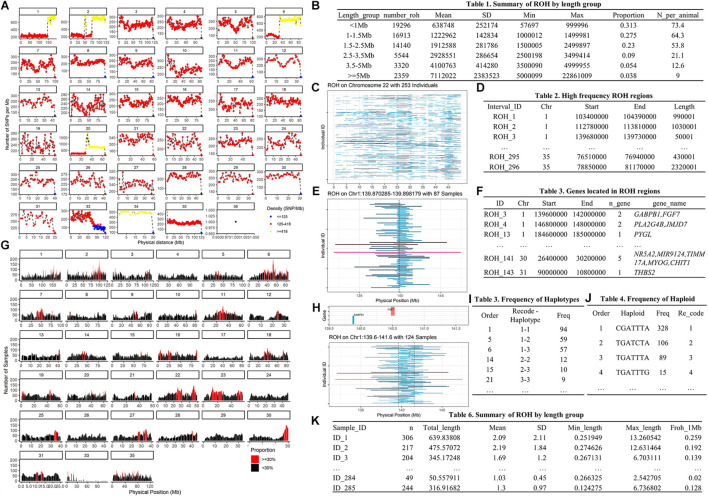
The main outputs of example 2. Panel **(A)** is SNP density distribution plot; panel **(B)** is brief summary of ROH by length group; panel **(C)** is plot of ROH on Chromosome 22; panel **(D)** is the high frequency ROH regions list; panel **(E)** is plot of ROHs on Chr1:139.6-141.6 Mb; panel **(F)** is genes annotation list of ROH regions; panel **(G)** is the ROH frequency distribution plot; panel **(H)** is plot of ROHs that overlap to the *GABPB1* gene; panel **(I)** is the frequency of haplotypes on *GABPB1* Gene; panel **(J)** is the frequency of haploids on the *GABPB1* gene; and panel **(K)** is the list of ROHs-based inbreeding coefficient.

To run this example, we first need to prepare the genotype data. The genotype files are read using the *fread* function (Dowle et al., 2019). Because the original ped file does not match the format required by Plink 1.9, we insert a sequential column of family IDs, plus placeholder columns of zeroes for the father, mother, and sex code by using *data.frame* and *cbind* functions (R Core Team, 2020). Before testing the ROH, the map file was loaded as the input file in *plot_snp_density* to get a brief summary and visualization of SNP density (Figure 7A). The *jpeg* and *dev.off* functions (R Core Team, 2020) are used to save the plot.

Then, we invoke Plink 1.9 (Chang et al., 2015) by *shell* (R Core Team, 2020) from R Studio (Team, 2021) to generate binary genotype files and call ROH. For Windows operating systems, ensure that the plink.exe file is either in the current directory or accessible via the PATH system variable. To run Plink 1.9 on other operation system, please refer to the Plink website (Chang et al., 2015).

Once we get ROH results, we can run *roh_window*, which takes a “plink.hom” file as input to report the brief summary of ROH by length group (see Figure 7B), high frequency ROH regions (see Figure 7D), ROH frequency distribution plot (see Figure 7G), and to calculate the ROH based inbreeding coefficient (Figure 7K).

In this example, we present visualizations of ROH on the whole of chromosome 22 (see Figure 7C) and on the 22.81–23.22 Mb region on chromosome 22 (see Figure 7E) via *roh_visual*, which needs to load the “plink.hom” data set as input. The “chr_id” or “target_region” arguments are available to customize visualization, alongside additional arguments to customize the colors of ROHs.

The horse reference gene list (“quaCab2”) was downloaded from the UCSC website (Navarro Gonzalez et al., 2021) by *get_refgene*. The genes located in the high frequency ROH regions (see Figure 7F) were annotated via *call_gene*, which requires loading the reference gene list (“quaCab2”) and the high frequency ROH regions file that was generated by *roh_window*. Since we have the reference gene list, we can visualize ROH region with genes (see Figure 7H) via roh_visual by assigning the clean ROH file (“clean_roh = clean_roh”), target ROH region [“target_region = c (1, 139.6, 141.6)”] and reference gene lists (“refgene = equaCab2”). We can also visualize ROHs in terms of the gene we are interested in: here, we are looking at the *GABPB1* gene, first, exacting the physical position of this gene from the reference gene list (“equaCab2”) using the “*filter*” and “*select*” functions (Wickham et al., 2019), then using *visual_roh* to load the ROH file (“plink.hom”) as input and assigning the gene position to the “target_region” argument to present the plot (see Figure 7E). We can write a loop (R Core Team, 2020) of *visual_roh* to plot all regions with genes annotated by iterating over the high frequency ROHs that contain genes.

To get the haplotype of the genes need the phased genotype files. Here, we take chromosome 1 as example to present how to use Plink 1.9 (Chang et al., 2015) and Beagle 5.1 (Browning et al., 2018) to phase the genotypes. The *shell* (R Core Team, 2020) function is used to invoke plink (Chang et al., 2015) to generate the VCF format genotype file, then to invoke beagle (Browning et al., 2018) to phase the genotypes from Rstudio (Team, 2021). For Windows operating systems, ensure that the plink and java executables are either in the current directory or accessible via the PATH system variable. Likewise, adjust the path to the Beagle JAR file as required for your operating system. For instructions on installing and running Beagle 5.1, refer to their manual (Browning et al., 2018).

Finally, we take *GABPB1* as an example to show how to get the haplotypes. First, we use *prep_phased* to load the phased genotype file (phased_geno = “orse_chr1_phased.vcf.gz”) that was generated by Beagle, and set the “convert_letter” argument as “TRUE” to convert the genotype file into the standard format used by HandyCNV (returned as “geno_chr1”). Second, we use *closer_snp* to extract the gene’s position (returned as “GABPB1_pos”) from the SNP map file, which requires the SNP map file (provided using the “phased_input” argument), and to assign the gene’s physical position we got from reference gene list to the “chr,” “start,” and “end” arguments, respectively. Finally, we use *get_haplotype* to get the haplotype information (see Figures 7I,J) for the *GABPB1* gene by assigning the formatted phased genotype list (“geno_chr1”) to the “geno” argument and assigning the gene’s position (“GABPB1_pos”) to the “pos” argument.

## Discussion

Here we present a freely available and open source R package called HandyCNV, which provides a comprehensive set of functions to summarize and visualize the CNVs and run of homozygosity results detected from SNP genotyping data.

Many good software packages have been developed for the detection of CNV and ROH from SNP chip data [such as PennCNV (Wang et al., 2007), CNVPartition (Illumina, 2021), SNP and Variation Suite (Bozeman and Golden Helix, 2020), and Plink (Chang et al., 2015)], and some well-designed tools for CNV-based association analysis [such as CNVRuler (Kim et al., 2012), CNVRanger (da Silva et al., 2019), and CNVassoc (Subirana et al., 2011)]. However, while they do include some basic data summary and visualization functions, they do not contain any features to customize visualization of CNV or ROH results, or to report the haplotype information for target genomic regions. In contrast to these tools, the HandyCNV package is focused on the detailed summarization and custom visualization of CNV and ROH results, facilitating tasks such as converting SNP maps, identifying CNVRs from lists of CNVs, genome annotation, comparing and visualizing CNV, CNVR, and ROH, reporting summary results and processing haplotypes of genomic regions of interest. The integration of multiple tasks into a single package provides a standardizable, reproducible and timesaving post-analysis of CNV and ROH, which can help researchers to produce comprehensive tables and figures, and easily identify the samples that contains the genomic regions or genes of most interest for the further validation of experiment designs.

There are some limitations to this package. For example, the *plot_cnvr_panorama* function needs to read genotype data to plot BAF and LRR information: this can require larger amounts of storage. We have tested it on 150 k SNP chip with 2,100 samples on a desktop windows system and it performs well; however, it may not be suitable for higher density chips and very large data sets. The *get_haplotype* function is also limited, as it currently only accepts phased genotypes produced by Beagle 5.1 (Browning et al., 2018) with physical position. In addition, the functions in the conversion section require users provide the target and default map files.

## Software Information

The current release of HandyCNV is version 1.1.6, which can be installed in the R environment using the following code: “remotes::install_github (repo = ‘JH-Zhou/HandyCNV@v.1.1.6’).” The current development version can be found at the GitHub repository (github.com/JH-Zhou/HandyCNV).

## Data Availability Statement

Publicly available datasets were analyzed in this study. This data can be found here: The human CNV lists used in Example 1 can be found in “Table S1 – Detailed information about all CNVs analyzed” at Supplementary Material section in Victória Cabral Silveira Monteiro de Godoy’s study (doi: 10.1590/1678-4685-GMB-2019-0218). The genotype data used in Example 2 can be found in Brandon D. Velie’s study which was public available via Figshare (doi: 10.6084/m9.figshare.3145759).

## Ethics Statement

Ethical review and approval was not required for the study on human participants in accordance with the local legislation and institutional requirements. Written informed consent for participation was not required for this study in accordance with the national legislation and the institutional requirements. Ethical review and approval was not required for the animal study because no animal sampling, experiments or phenotype measurement applied in this study. The genotype data used in this analysis are from previous studies.

## Author Contributions

JZ conceived the analysis, compiled the package, and wrote the manuscript. LL contributed to code writing and testing, and reviewed the manuscript. TL contributed to package testing, proofreading of the manuscript, and vignette. DG and YS provided instruction for analysis, reviewed the manuscript, manual, and vignette. All authors contributed to the article and approved the submitted version.

## Conflict of Interest

TL is employed by Livestock Improvement Corporation. The remaining authors declare that the research was conducted in the absence of any commercial or financial relationships that could be construed as a potential conflict of interest.

## Publisher’s Note

All claims expressed in this article are solely those of the authors and do not necessarily represent those of their affiliated organizations, or those of the publisher, the editors and the reviewers. Any product that may be evaluated in this article, or claim that may be made by its manufacturer, is not guaranteed or endorsed by the publisher.
